# MASSIVE COMMON BILE DUCT GANGRENE OF OBSCURE ETIOLOGY ENCOMPASSING THE ENTIRE HEPATO-DUODENAL LIGAMENT IN A CLINICALLY SILENT ADULT: WORLD’S FIRST CASE

**DOI:** 10.1590/0102-672020190004e1565

**Published:** 2021-05-14

**Authors:** Priyadarshan Anand JATEGAONKAR, Sudeep Pradeep YADAV, Jinit Salil SHAH, Piyush Raghunath DHAIGUDE, Aditi Rajeev SOOD

**Affiliations:** 1 Department of Surgery, Division of GI & Minimal Access Surgery, Mahatma Gandhi Institute of Medical Sciences, Sevagram, Wardha- 442102, Maharashtra, India; 2 Department of Plastic & Reconstructive Surgery, Grant’s Government Medical College & Sir J.J. Group of Hospitals, J.J. Marg, Nagpada, Mumbai Central, Off Jijabhoy Road, Mumbai-400008, Maharashtra, India; 3 Department of Surgery, Mahatma Gandhi Institute of Medical Sciences, Sevagram, Wardha- 442102, Maharashtra, India; 4 Department of Radiology, Mahatma Gandhi Institute of Medical Sciences, Sevagram, Wardha- 442102, Maharashtra, India

**Keywords:** Common bile duct, Gangrene, Biloma, Perforation, Peritonitis, Ducto biliar comum, Gangrena, Bilioma, Perfuração, Peritonite

## INTRODUCTION

Common bile duct (CBD) gangrene is an extremely rare cause of spontaneous biliary perforation leading to life-threatening biliary peritonitis^3^. Only one such case has been reported, way back in the year 1951^3^. Herein, we present an unusual case of massive CBD gangrene that also involved hepato-duodenal ligament. Moreover, we propose an algorithm for its preoperative diagnosis. To our knowledge, such a case is yet to be reported.

## CASE REPORT

**FIGURE 1:**
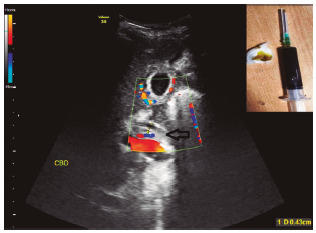
Duplex ultrasonography showing small-caliber CBD lacking adequate vascularity

Note: The segmental loss of CBD is responsible for bile extravasation (arrow); inset: abdominal paracentesis sample depicting frank watery bile without any residue. The bile’s apparent green color (syringe), is actually a golden-yellow when smeared (cotton swab)-typical of CBD origin; CBD=common bile duct

A 52-year-old male was admitted under the Department of Internal Medicine at our rural academic institute for evaluation of ascites and was referred to us for surgical consultation for his recent-onset painless jaundice. Chronic alcoholic since15 years, he had noticed progressive abdominal distension and yellowness of his sclera over three days. He denied any recent abdominal pain, fever or trauma. With regular bowel habits, he passed yellow-brown stools and had no medical co-morbidities. On examination, he had normal vital parameters, was mildly icteric, and had no signs of chronic liver failure. His abdomen was uniformly distended, non-tender, with no organomegaly. Positive shifting dullness confirmed presence of free fluid in his abdomen. The hematological screen, except for raised serum total bilirubin (3.4 mg/dl), was within normal limits. His erect chest radiogram showed no free air under the diaphragms. Abdominal ultrasonography demonstrated gross free fluid without any internal echoes or septations. His gallbladder appeared distended with no luminal stones or pericholecystic fluid. His liver, intra-hepatic biliary radicles and pancreas were normal. On addition of color Doppler, the CBD illustrated compromised vascularity with incomplete circumferential visualization (Figure 1). Diagnostic paracentesis was helpful^1^ revealing yellowish-green bile having watery consistency without any food-debris, mucus or pus flecks (Figure 1). Sterile on culture, the ratio of its bilirubin content to that of the serum was as high as 10:1, with negligible amylase level. After adequate resuscitation he underwent emergency exploratory laparotomy. 

**FIGURE 2:**
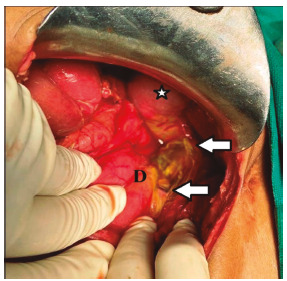
Operative photography depicting gangrenous hepato-duodenal ligament containing sloughed CBD with hardly any healthy remnant

Note: The cranial and caudal margins of the gangrenous process (white arrows) extend from the normal gallbladder (*) to the healthy duodenum (D); also note, the golden-yellow bile from the disrupted CBD getting pooled into the Morrison’s pouch without staining the retroperitoneal tissue-planes or bowel, implies an acute biliary insult; CBD=common bile duct

At surgery, about 2.5 liters of non-turbid bile poured from the peritoneal cavity. The gallbladder, the entire bowel and the pancreas were all healthy, and the lesser sac was empty. Also, the whole hepato-duodenal ligament along with the CBD was gangrenous and continuously leaked golden-yellow bile (Figure 2). Here, any attempts to probe such a paper-thin CBD were restrained for avoiding further damage. The friable sub-hepatic tissues along with the impending ionotropic support discouraged even duodenal Kocher maneuver and signified “primum non nocere”^2^ ^,^ ^5^. Rigorous peritoneal lavage was followed by a drain each in the Morrison’s pouch and in the pelvis before abdominal closure^2^.

**FIGURE 3:**
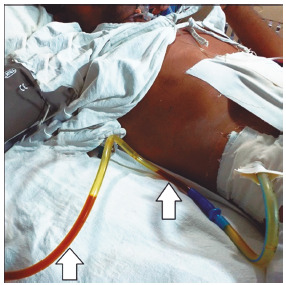
Clinical picture on postoperative day 11 illustrating a low-output controlled external biliary fistula

Note: The sub-hepatic drain containing clear golden-yellow bile (white arrows) clinically confirms its source as the CBD; also note, the patient’s scaphoid abdomen without any skin excoriation largely indicates a satisfactory surgical outcome; CBD=common bile duct

With dramatic postoperative recovery, his jaundice subsided and the serum bilirubin level returned to the standard by day-3. He passed regular stools on 4^th^ day and tolerated oral diet thereupon. As expected, his sub-hepatic drain yielded 200-300 ml typical golden-yellow bile everyday till day 11 (Figure 3). 

**FIGURE 4:**
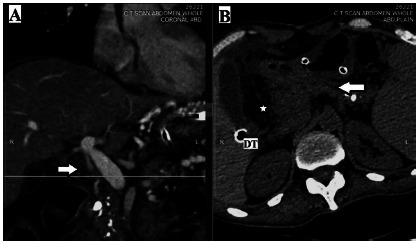
CECT abdomen on postoperative day 11: A) coronal section: non-enhancing and completely collapsed CBD (white arrow) represents its ischemia and perforation, respectively; B) sagittal section: normal pancreas and non-dilated main pancreatic duct (white arrow) excludes obstructive calculus as a cause of CBD perforation.

Note: Except for the minimal residual fluid (*) around the drain-tube (DT) placed in the Morrison’s pouch, the rest of the peritoneal cavity is clean; CECT=contrast- enhanced computer tomogram; CBD=common bile duct

A check abdominal contrast-enhanced computer tomogram (CECT) depicted a non-enhancing CBD having patchy mural loss without any calculi in its lumen or in the peritoneal cavity. Its axial blood vessels could not be delineated. The pancreas and its duct were normal, and both the drain-tubes were in situ. There were no residual fluid collections, and the oral contrast confirmed absence of duodenal perforation (Figure 4). 

After optimizing his nutritional and performance status over three weeks, he underwent definitive surgery in the form of CBD excision followed by Roux-en-Y hepaticojejunostomy and had satisfactory recovery thereafter^1^ ^,^ ^4^.

## DISCUSSION

Our case has several notable learning points: 1) massive CBD gangrene with its near-total disruption to cause choleperitoneum, when most of the reported perforations are limited to <1 cm^3,^ ^5^; 2) possible involvement of entire hepatoduodenal ligament in the gangrenous process which has not been reported so far; 3) clinical silence despite the severity for over three days; 4) affecting an adult at a relatively older age^1^ ^,^ ^2^ ^,^ ^4^; and 5) mural ischemia due to blockage of the precarious axial biliary vessels, when the commonest cause reported is choledocholithiasis^1^ ^,^ ^5^.

Despite significant advances in diagnostic modalities, biliary gangrene usually arrives as a delayed per-operative diagnosis, often revealed as an unpleasant surprise^1^ ^,^ ^4^. This could lead to adverse surgical and socioeconomical outcomes^1^ ^,^ ^2^. However, in our case, a flawless preoperative diagnosis could be attained by the following thought- spiral: physical and biochemical characteristics of the sampled bile in absence of any clinical or radiological signs of peritonitis contemplated a sterile choleperitoneum, most likely originating from the extra-hepatic biliary tree; the morphology of gallbladder on ultrasonography ruled out gallbladder perforation and prompted us to add color Doppler to assess the possibility of biliary gangrene; it was confirmed at surgery.

Absence of biliary lithiasis on abdominal ultrasonography, normal liver enzymes, yellow stools and lack of colicky upper abdominal pain practically ruled out choledocholithiasis as the cause of his jaundice and CBD perforation. It also implied brisk trans-peritoneal bilirubin absorption as the mechanism for his icterus. By this approach, we could defer expensive and scarcely available radiological tests like CECT or magnetic resonance imaging without compromising the management policies; this could be of great concern at resource-limited settings. Postoperative external biliary fistula showing clear golden-yellow bile with negligible amylase further corroborated CBD pathology. And, its low-output nature suggested biliary-enteric continuity. Finally, postoperative CECT substantiated our diagnostic algorithm.

However, as the exact cause of this vascular insult could not be identified, it was deemed idiopathic^5^.
